# Hemorrhoidal artery ligation with Doppler guidance vs digital guidance for grade II-III hemorrhoidal disease treatment: Study protocol clinical trial (SPIRIT Compliant)

**DOI:** 10.1097/MD.0000000000019424

**Published:** 2020-04-10

**Authors:** Daniil Markaryan, Inna Tulina, Tatiana Garmanova, Mikhail Bredikhin, Aftandil Alikperzade, Petr Tsarkov

**Affiliations:** Federal State Autonomous Educational Institution of Higher Education I.M. Sechenov First Moscow State Medical University of the Ministry of Health of the Russian Federation (Sechenov University), Moscow, Russia.

**Keywords:** Doppler-guided ligation, finger-guided artery ligation, hemorrhoids, hemorrhoidal piles prolapse

## Abstract

**Introduction:**

Hemorrhoidal artery ligation (HAL) with Doppler guidance and suture fixation of hemorrhoidal nodes (RAR) is a popular minimally invasive technique for hemorrhoidal disease (HD) treatment which uses an ultrasound probe to detect hemorrhoidal arteries for further ligation. We hypothesized that ultrasound guidance has no advantages over manual hemorrhoidal arteries detection for HD treatment.

The aim is to compare the results of HAL-RAR procedure in patients with stage II-III HD with Doppler and manual HA detection.

In this ongoing randomized, controlled, single center clinical study 204 patients randomly divides into group A (HAL-RAR with Doppler US navigation) and group B (HAL with manual HA detection and mucopexy) are planned to be included. The primary endpoint was recurrence of any symptoms of HD; secondary endpoints were pain syndrome severity, treatment satisfaction (1 to 5 points), and need for the drug therapy in 30 days and 8 weeks after surgery.

**Conclusion:**

Ultrasound guidance technology of HAL with mucopexy could have the same efficacy the manual HA detection regarding the HD treatment effectiveness and patient satisfaction.

## Abstract 2

Supplemental Digital Content is available in the text

## 1 Introduction

### 1.1 Background and rationale

Hemorrhoidal disease (HD), in its different manifestations, is not only the most frequented grounds of referring for medical attention, but also one of the reasons for the modest deterioration in the quality of life (QOL) that can possibly result in temporary or permanent reduction of work capacities. Today, the Doppler-guided dearterialization of hemorrhoidal arteries and the following suture-fixation mucopexy in the anal canal (synonyms: mucopexy, hemorrhoids lifting, hemorrhoidal artery ligation–suture fixation of hemorrhoidal nodes [HAL-RAR]) is one of the most popular and actively studied methods of the stage II-III HD surgical treatment. A number of publications raise an issue whether it is really necessary to use a Doppler while the localization of the hemorrhoidal arteries is typical in the vast majority of the observations and can be easily determined by palpation.^1^,^2^,^3^,^4^

### 1.2 Objectives

#### 1.2.1 Research hypothesis

The digital detection of hemorrhoidal arteries pulsation followed by suture ligation and mucopexy may be at least as effective in the treatment of grade II-III hemorrhoids as the Doppler-guided dearterialization of hemorrhoidal arteries.

#### 1.2.2 Study objectives

##### 1.2.2.1 Primary objective

To determine if the palpation-guided hemorrhoidal arteries ligation and mucopexy is noninferior to Doppler-guided ligation of hemorrhoidal arteries and mucopexy in patients with II-III grade HD at the endpoint of recurrence rate determined by physical examination and patients complaints.

##### 1.2.2.2 Secondary objectives

Key secondary objectives:

1. Overall complication rate.
2. Patient-reported pain level using visual scale in 1 and 2 months after surgery.
3. Patients’ satisfaction rate using a 10-points scale in 6 month and 1 year after surgery.

### 1.3 Trial design

HAND study is a prospective, randomized, controlled, unicenter, noninferiority, parallel group, 2-arm trial with 1:1 allocation ratio. Randomization will be performed using block method before the procedure.

## 2 Methods

### 2.1 Study setting

The study will be conducted at the Clinic of Colorectal and Minimally Invasive Surgery of the Sechenov University Hospital N. 2 in Moscow, Russia. Patients aged form 18 to 75 years of all genders with symptomatic hemorrhoids grade II and III according to Goligher classification will be enrolled in this clinical trial. Thus, in this case, a single-center design can assure sufficient patient recruitment.

### 2.2 Eligibility criteria

To be eligible for randomization, an individual must comply with all of the following criteria:

Inclusion criteria:

- Symptomatic grade II and III hemorrhoids according to Goligher classification.
- No other source of anal bleeding than hemorrhoids due to total colonoscopy.
- Written voluntary informed consent.

Exclusion criteria:

- Any previous hemorrhoid surgery (including mini invasive procedures).
- Anal fistula.
- Chronic anal fissure with severe spasm of anal sphincters.
- Any stage colorectal cancer.
- Oral anticoagulants for congenital disorders of the coagulation system.
- Pregnancy.

### 2.3 Interventions

#### 2.3.1 Preoperative preparation

No special preparation is required before procedure.

#### 2.3.2 Day of surgery

Under a spinal anesthesia the patient is placed in a modified lithotomy position on the back, with legs spread apart on supports. The operative field is treated with an antiseptic solution twice and draped.

##### 2.3.2.1 Surgical technique

Doppler-guided HAL will be performed as following: a lubricating gel is applied to the tip of the transanal hemorrhoidal dearterialization device and, with the patient in the lithotomy position, the proctoscope is introduced into the anal canal. The terminal branches of the superior rectal artery are detected by the Doppler signal 2 to 3 cm above the dentate line. The tip of the instrument is gently tilted and the arteries are ligated with a Z-shaped stitch using 2:0 braided polyglycolic acid suture inserted using a special needle holder through an aperture in the operating proctoscope. Mucopexy: after the hemorrhoid artery ligation, the suture is continued with 3 to 5 sutures applied 5 mm apart, making sure that the last is at least 5 mm above the dentate line. The suture is then tied to create a hemorrhoidopexy. The procedure is repeated after all detected artery ligations.

Finger-guided ligation of hemorrhoidal arteries without Doppler guidance will be performed as following: the exact placement of all terminal branches of the superior rectal artery are found by intraoperative palpation at anal clock 2 to 3 cm above the anorectal junction. Then arteries are ligated with Z-shaped suture using 2:0 braided polyglycolic sutures. Mucopexy is then performed in the same technique as in comparative group

#### 2.3.3 Early postoperative period

It is defined as the time period from the completion of the surgery and up to 30 days after the surgery. An obligatory regimen of analgesia will be prescribed for each patient, which includes nonsteroidal analgesics, paracetamol, and topical symptomatic drugs. No diet before or after the surgery. The patient will be discharged form hospital after the first defecation if no further medical care is needed.

#### 2.3.4 Late postoperative period

It is defined as the period starting at 31st day post surgery. The patient is asked to fill out the questionnaire forms to assess the changes in the QOL and pain level after surgery. To ensure patient compliance to protocol requirements, all subjects will be notified telephonically and/or via e-mail. If the patient is unavailable, they must be contacted at least 3 times within the follow-up timeframe, before considered dropped-out from the study.

### 2.4 Outcomes

#### 2.4.1 Primary outcome measure

The recurrence of HD is determined as presence any of initial symptoms or appearance of any new symptom of HD: anal bleeding during defecation, prolapse of hemorrhoidal piles, chronic pain, or their combinations.

#### 2.4.2 Secondary outcome measures

1. The pain score after surgery will be measured by patient-reported pain level using visual scale ranging from 1 to 10 where 1 is “no pain" and 10 is the worst pain imaginable in 1 and 2 months after surgery.
2. QOL assessment (patients satisfaction level): Patients will be asked to rate their own satisfaction of the procedure on a scale from 1 to 10 (with 10 being the best) and were asked whether the procedure helped their symptoms in 6 month and 1 year after surgery.
3. Early postoperative complications rate (time frame: 30 days): The rate of complications in 30 days after surgery.

### 2.5 Sample size

Considering that this is a noninferiority study, the sample size was calculated using 1-sided Blackwelder test.^[5]^ According to published data, the incidence of recurrence rate of suture ligation of the hemorrhoidal arteries with mucopexy varies from 20% to 30%.^1^,^2^,^3^,^4^ The expected incidence of recurrence rate of HD after digital detection of hemorrhoidal arteries pulsation followed by suture ligation and mucopexy is not more than 35%. The purpose of this study is to show that the outcomes of HAL-RAR procedure with digital detection of hemorrhoidal arteries is noninferior compared to HAL-RAR procedure with Doppler-guided hemorrhoidal arteries detection.

Considering that a = 0.05; the statistical power of the study is 80%; the patients are randomized into 2 groups with 1:1 allocation ratio; the noninferiority margin D = 5%, the required sample size is 204 patients (102 patients in each of the 2 groups).

### 2.6 Recruitment

All patients diagnosed with HD II-III stage will be considered for this study.

### 2.7 Assignment of interventions

#### 2.7.1 Allocation

Participants will be randomly assigned to either control or experimental group with a 1:1 allocation ratio using fixed block randomization with a computerized random number generator. All subjects will be allocated preoperatively.

#### 2.7.2 Blinding

All relevant data from patient chart except patients’ names and the stapler used during surgery will be transferred into an electronic case report form (eCRF). The eCRF should contain results of all the screening procedures, including patient history and demographics, imaging studies, filled-out questionnaires, operation note, and postoperative rounds during patients’ stay in the surgical ward.

### 2.8 Data collection, management, and analysis

#### 2.8.1 Data collection methods

All data will be collected prospectively using eCRFs designed for this trial. The reasons for withdrawal will be documented. The investigator will attempt to contact each participant at least 3 times during each follow-up window before declaring them lost for observation. The study exit form will be recorded in the eCRF. All prior data will be analyzed within the research.

#### 2.8.2 Data management

All patients will receive clarifications of all the study procedures, and will be able to discuss them with the primary investigator. All patient data will be handled according to the principles of doctor–patient confidentiality; the subjects will be anonymized and analyzed with individual identifier numbers transcribed into eCRF.

#### 2.8.3 Statistical methods

An intention-to-treat analysis will be performed. Statistical analysis was carried out using IBM SPSS Statistics v23 software (IBM Corporation, US, New York), and included a comparison of categorical (Fisher exact test) and quantitative (unpaired Student *t* test) indicators in groups. The results were considered statistically significant, with a value of *P* ≤ .05.

#### 2.8.4 Data monitoring

There is no data monitoring committee designated to this trial. Any adverse and serious adverse events will be immediately reported to the principal investigator and the primary sponsor.

### 2.9 Ethics and dissemination

#### 2.9.1 Research ethics approval

This study follows the Declaration of Helsinki on medical protocols and ethics; all documents of the trial have been approved by the local Ethics Committee of Federal State Autonomous Educational Institution of Higher Education I.M. Sechenov First Moscow State Medical University of the Ministry of Health of the Russian Federation (Sechenov University) (reference no. 10–19).

#### 2.9.2 Protocol amendments

Any protocol amendments that may influence the conduct of the study, will be communicated to the local ethics committee and study director, and will be uploaded to clinical trials.

#### 2.9.3 Consent or assent

A member of the research team will obtain the consent form. All participants will be able to address their questions about the study to 1 of the members of the research team. Consent form is described in Supplemental Digital Content (Appendix 1).

#### 2.9.4 Confidentiality

All patient data will be secured at the study site. No one apart from the members of the research team will have access to any patient data, including anonymized eCRFs with a coded ID, as well as filled out questionnaires.

#### 2.9.5 Access to data

No one apart from the members of the research team will have access to the final trial dataset.

#### 2.9.6 Dissemination policy

Trial results will be e-mailed to all participants of the trial. Trial results will be disseminated to healthcare professionals via publication in a peer-reviewed scientific journal and by mass media, as well as conference papers to inform the public and stakeholders, and will be uploaded to the primary registry. We have no intention of granting public access to the full protocol, participant-level dataset, and statistical code.

## 3 Discussion

The results of this RCT may demonstrate the absence of significant advantages in Doppler guidance technology for hemorrhoidal arteries detection in HAL-RAR procedures compared with the simple and accessible to any surgeon finger-guided hemorrhoidal arteries detection concerning the efficiency in eliminating hemorrhoidal prolapse and/or hemorrhoidal bleeding. We will analyze frequency of postoperative complications, the intensity of the pain syndrome, the relapse rate, and individual QOL level. It will allow us to conclude whether the HAL-RAR with finger hemorrhoidal artery detection is effective and safety procedure for hemorrhoids disease treatment.

The design of this study has certain limitations. Firstly, there is quite often mismatching of doctors regarding the stage of the HD as well as stage of each hemorrhoidal pile. So the group might be heterogenic regarding the preoperative stage of the HD.

Another issue is the uncertainty of the definition of HD recurrence after surgery. In this protocol, we determined it as appearance of any new symptoms of HD or persistence of previous ones. But we suppose that nevertheless procedure might be effective for preoperatively existed symptoms new ones might appear. So we will try to figure out the difference of preexisting symptoms resolution and appearance of new ones.

## Acknowledgment

The authors express their gratitude to Sechenov University administration for their support of this work.

## Author contributions

All listed authors fulfill the authorship criteria defined by the International Committee of Medical Journal Editors.DM, PT, and TG participated in the conception and design of the study, MB drafted the current manuscript and performed statistical analysis. DM, TG, PT critically reviewed the manuscript, AA, IT were responsible for project administration andvisual content. All authors read and approved the final manuscript.

**Conceptualization:** Petr Tsarkov and Daniil Markaryan.

**Data curation:** Mikhail Bredikhin, Tatiana Garmanova.

**Project administration:** Petr Tsarkov.

**Resources:** Aftandil Alikperzade.

**Software:** Mikhail Bredikhin.

**Supervision:** Petr V. Tsarkov.

**Writing – original draft:** Tatiana Garmanova, Daniil Markaryan.

**Writing – review & editing:** Petr V. Tsarkov.

Tatiana Garmanova orcid: 0000-0003-2330-4229.

## Supplementary Material

Supplemental Digital Content
