# Supplementary material for: Hemorrhoidal artery ligation with Doppler guidance vs digital guidance for grade II-III hemorrhoidal disease treatment: Study protocol clinical trial (SPIRIT Compliant)
Source: Medicine (Baltimore). 2020 Apr 10;99(15):e19424. doi: 10.1097/MD.0000000000019424 (PMC7220052; doi:10.1097/MD.0000000000019424)
Supplement: Supplemental Digital Content [file medi-99-e19424-s001.docx]

Supplemental Digital Content (Appendix 1)

Patient informed consent

By signing this informed consent form, you are giving your full consent to the collection, recording, storage, transfer or disclosure of your medical information. You understand that you will not be able to participate in research, if you do not give your consent to the collection and use of your medical information.

If you have cause for concern or complaints related to research or its implementation, please feel free to discuss them with your research physician.

If you are not comfortable discussing your problems with your

research doctor, you have the right to contact the chief research doctor:

FULL NAME: ________________________________________________________________

Address: ___________________________________________________________

Phone: ___________________________________________________________

If you want to know more about your rights as a research participant,

or would you like information or share it, or would you like to speak with

by someone not directly related to the study, you can contact:

To a member of the Independent Ethics Committee:

Name: ___________________________________________________________

Address: ___________________________________________________________

Name of contact person: ________________________________________________

Phone number: _____________________________________________________

1. I have read and understood the information provided in this Informed patient consent

2. I had the opportunity to ask questions, and for all my questions I received comprehensive answers.

3. I voluntarily agree to participate in this study.

4. I do not waive my legal rights by signing this document.

5. The signature on this document means that I agree to participate in research. I acknowledge that at any time I can stop participating in research.

6. I understand that, if I agree, I will receive a signed and dated copy of this document.

7. I give my consent to the processing, collection and storage of my confidential

data and information from the medical record

___________________________ _____________ ____________

Name Patient Block Letter Date Signature

___________________________ _____________ ____________

Name in block letters of the doctor, date Signature

negotiator of consent *
